# Toxicity mitigation by *N*-acetylcysteine and synergistic toxic effect of nano and bulk ZnO to *Panagrellus redivivus*

**DOI:** 10.1007/s11356-021-12674-7

**Published:** 2021-03-02

**Authors:** Lola Virág Kiss, Zoltán Sávoly, András Ács, Anikó Seres, Péter István Nagy

**Affiliations:** 1 Department of Zoology and Animal Ecology, Szent István University, Gödöllő, Hungary; 2 Budapest, Hungary; 3 Department of Aquaculture, Institute for Conservation of Natural Resources, Faculty of Agricultural and Environmental Sciences, Szent István University, Gödöllő, Hungary

**Keywords:** Zinc-oxide, *N*-acetylcysteine, Interaction, Nanomaterial, Nematode, Synergistic

## Abstract

To better understand the nanosize-relevant toxic effects and underlying mechanisms, *N*-acetylcysteine (NAC), as a mitigation agent, an ionic form of Zn (ZnCl_2_), and the binary mixture of ZnO with different particle sizes (15 nm and 140 nm), was used in toxicity assays with the nematode *Panagrellus redivivus*. The ZnCl_2_ concentrations were applied to show the amount of dissolved Zn ions present in the test system. Reactive oxygen species (ROS) measuring method was developed to fit the used test system. Our studies have shown that NAC can mitigate the toxic effects of both studied particle sizes. In the applied concentrations, ZnCl_2_ was less toxic than both of the ZnO particles. This finding indicates that not only ions and ROS produced by the dissolution are behind the toxic effects of the ZnO NPs, but also other particle size-dependent toxic effects, like the spontaneous ROS generation, are also relevant. When the two materials were applied in binary mixtures, the toxic effects increased significantly, and the dissolved zinc content and the ROS generation also increased. It is assumed that the chemical and physical properties of the materials have been mutually reinforcing to form a more reactive mixture that is more toxic to the *P. redivivus* test organism. Our findings demonstrate the importance of using mitigation agent and mixtures to evaluate the size-dependent toxicity of the ZnO.

**Supplementary Information:**

The online version contains supplementary material available at 10.1007/s11356-021-12674-7.

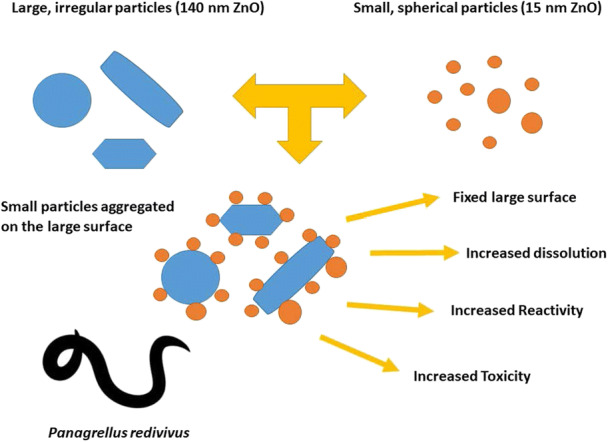

## Introduction

The prevalent use of nanotechnology in each part of human life results in an immense release of engineered nanomaterials to the environment. Some metal-based nanomaterials like zinc oxide (ZnO) reached significant importance for their extensive commercial applications. According to Coll et al. (2016) and Kiss et al. (2020), ZnO is the nanomaterial of the greatest concern because of its exhibited high toxicity and predicted exposure concentrations.

After being released to the soil environment, ZnO NPs (zinc-oxide nanoparticles) can be adsorbed onto soil particles, react with organic materials, and even be transported to groundwater. As a result of its increasing released amounts (Gottschalk et al. 2009, Sun et al. 2016) and its potential leaking into pore water, ZnO can pose a risk to soil microfauna, including nematodes. Soil living nematodes are highly exposed to ZnO NP pollution in the environment. There is vast evidence in the scientific literature showing that ZnO NPs are toxic in the applied concentrations (Ma et al. 2011, Wu et al. 2013, Khare et al. 2014, Sávoly et al. 2016, Kiss et al. 2018). Several studies observed significant mortality trends for various nematode species at concentrations as low as 0.32–0.65 mg/l (Khare et al. 2011, Kiss et al. 2018). Nanosize-relevant toxicity varied from laboratory to laboratory. Different test methods and the physical and chemical differences in the materials used may have been lied behind that. In most of the cases, however, ZnO NPs with smaller particle size were more toxic (Ma et al. 2011, Khare et al. 2011). Free-living soil nematodes are part of the food chain, are involved in degradation and remediation processes and have great importance in the global biogeochemical cycles of various substances (Vinciguerra 1979, Sochová et al. 2006). Therefore, it is crucial to investigate the effects on soil nematodes exposed directly to ZnO NPs, preferably in the most environmentally relevant test medium.

Due to their varied applicability, the release of ZnO NPs is expected to increase, despite the vast amount of data available on potential hazards and risks, making reducing the toxic effects of ZnO NPs a paramount task. A suitable method is to mitigate or even eliminate the negative effects in a way that beneficial properties are maintained, with the help of various mitigating agents. The *N*-acetylcysteine (NAC) antioxidant can be suitable for this task because NAC is considered an important antioxidant and free radical scavenger by increasing intracellular glutathione levels (Sun 2010) and downregulating AP-1 luciferase activity (Shi et al. 2017). Moreover, thiol groups in NAC reduce free radicals and also provide the chelating site for metals (Flora and Pachauri 2010). Due to these abilities, NAC has been widely used in several research fields for investigating the toxic effects of ZnO NPs (Ma et al. 2014, Wang et al. 2014, El-Shorbagy et al. 2019). Toxicity mitigation effects were successfully observed in the relation of nematodes tested with TiO_2_ NPs (Wu et al. 2012, 2013), Al_2_O_3_ NPs (Li et al. 2012), and also ZnO NPs (Wu et al. 2013). Reports about the environmental effects of NAC are quite scarce, and its effect on the biota is relatively unknown. No safety concerns were described in human and animal studies collected in Bhatti et al.’s (2018) review about the topic. Moreover, they reported reduced apoptosis and oxidative stress. However, NAC can be overdosed and cause severe health problem such as hemolysis, thrombocytopenia, and death (Mahmoudi et al. 2015). NAC also have antibacterial properties; it is able to disperse biofilms of both Gram-negative and Gram-positive bacteria (Nowacka et al. 2018).

The ZnO NP toxicity can result from multiple properties, including photo-induced and regular dissolution of zinc ions, generation of reactive oxygen species (ROS), and other potential particle-specific effects like direct contact between the particles and the cells of an organism (Brunner et al. 2006, Khare et al. 2014, Starnes et al. 2019). Moreover, size relevant effects of ZnO nanoparticles (NPs) and excess release of Zn^2+^ can also result in the generation of ROS. Even so, most studies point to ZnO NPs dissolution to ionic Zn playing the most significant role in eliciting toxicity (Ma et al. 2014, Wang et al. 2016, Sávoly et al. 2016, Lead et al. 2018).

The wide use of diverse engineered nanomaterials can also lead to the release of different mixtures of nanomaterials into the environment. However, little is known about the combined toxicity of various nanomaterials (Mott et al. 2007, Jafari et al. 2011, Li et al. 2011, Tong et al. 2014, 2015). No information was found on the combined toxicity of ZnO NPs and its bulk form, although exposure to such a complex is a realistic scenario as well. Furthermore, experiments dealing with a mixture of differently sized nanomaterials can also elucidate the size relevant effects of them.

In the present study, the main aim was to better understand the size relevant toxic effects of the ZnO on the nematode *Panagrellus redivivus* and mechanisms behind them. The usage of a mitigation agent, an ionic form of Zn, and a binary mixture of two ZnO agents with different sizes helped to explore size-dependent toxicity of the ZnO particles. In this research, we hypothesized that the nano-relevant toxic effects could be identified through mitigation assays with NAC, as it influences both the ionic and the ROS induced toxicity. It was assumed that ZnCl_2_ in the concentration series set by the dissolution rate of the ZnO compounds could elucidate the role of ionic toxicity in the toxicity mechanism of ZnO NPs. Furthermore, we hypothesized that the environmentally relevant combination of nano and bulk ZnO particles (15 and 140 nm) has different effects on used test species as compared with the same particles applied separately. For the aim of better clarifying the difference between compound effects, combined toxicity was also tested with the addition of NAC. In addition, we developed a technique to measure ROS production in a way that fits to the test system we used.

## Materials and methods

### Particle size characterization and sample preparation

Two ZnO particles of different nominal particle sizes were applied as (i) 10–30 nm (referred to as 15 nm average particle size) with purity 99+% and (ii) 80–200 nm (referred to as 140 average particle size) ZnO with purity 99.9+%. Both materials were purchased from US Research Nanomaterials, Inc. The particle shape of the 15 nm ZnO was nearly spherical, and that of the 140 nm ZnO was irregular. ZnCl_2_ was used as a free ionic positive control. For the mitigation experiments, *N*-acetylcysteine (NAC) was the mitigation agent. Both ZnCl_2_ and NAC were purchased from Sigma-Aldrich.

The primary particle size and particle morphology were measured by scanning electron microscopy (SEM, FEI Quanta 3D, Eötvös Loránd University, Hungary). The materials were examined individually in previous experiments (Kiss et al. 2018). Both ZnO compounds contained nano and bulk particles; however, based on the definition of nanomaterials*—*the particle size of at least half of the particles in the number size distribution must measure 100 nm or below*—*the 15 nm was in nanosize range and the 140 nm was in bulk size range (Kiss et al. 2018). Images were recorded from the binary mixture of the two, and the average size and size distribution were ascertained by measuring approximately 100 particles from representative images by the ImageJ software package. The ZnO particles were also checked with the addition of *N*-acetylcysteine after 24 h incubation by SEM.

New stock suspensions were made from the 15 and 140 nm and the binary mixture of them (referred to as Mix or mixture) (160.64 mg Zn/l) and ZnCl_2_ (67.68 mg Zn/l) powders with Milli-Q water before each experiment. The mixture contained an equal amount (50–50%) of ZnO particles with an average size of 15 and 140 nm, respectively. The stock suspensions were dispersed by sonication for 20 min every time. Tested concentrations were chosen based on our previous experiments (Kiss et al. 2018). Environmentally relevant concentration of ZnO NPs was about 1.52–21 μg/l in 2016 (Sun et al. 2016); however, this value is increasing day by day due to the huge amount of released ZnO NPs to the environment. The concentration series were prepared from the stock immediately after sonication and added to the test media. They were set up based on pre-tests of our research group. No lethal effects were observed in the environmentally relevant concentration range, so higher values were used to get the desirable effects.

### Dissolution of ZnO nanoparticles

The dissolution of materials in Milli-Q water (from 5 ml stock, 10.4 mg Zn/l) after 24 h of incubation time in a dark thermostat chamber was evaluated by centrifugation, followed by chemical analysis of complex supernatant zinc using inductively coupled plasma atomic emission spectrometry (ICP-AES, Horiba Jobin-Yvon Activa-M, SZIE, Hungary) (Ma et al. 2014). Dissolution was assessed for pure 15, 140 nm, mixture, and with the addition of NAC with all the mentioned compounds. Zn particles held in a complex by NAC were also included in the measurement.

### The mitigation effect of *N*-acetylcysteine and toxicity of ZnCl_2_ compared with ZnO NPs on *Panagrellus redivivus*

To facilitate the evaluation of results, an easily and quickly operated test system was chosen: a dose-response study with a bacterivore nematode, *Panagrellus redivivus. P. redivivus* can be found in a variety of nutrient-rich habitats such as soil, rotting fruits, insects, wheat paste, or beer yeast. It is an excellent species for studying the toxic effects of nanomaterials being easy to work with, having a high reproduction rate, and showing relatively minor differences from *Caenorhabditis* elegans, the most commonly used nematode species, in cell lineage (Sternberg and Horvitz 1982).

Toxicity assays were carried out based on modifications prompted by previously published methods (Ma et al. 2014, Hrács et al. 2018, Kiss et al. 2018). In a 96-well microplate (Bioster S.p.A., Italy), 5 adult nematode females were placed in each unit for testing acute mortality in Milli-Q water test media. The tests were performed with pure nano, bulk ZnO particles and ZnCl_2_, and with added NAC (5 mg/l concentration). The used NAC concentration was based on previous research of Ma et al. (2014) and Wu et al. (2013). Applied concentrations for ZnO and ZnCl_2_ compounds were 0.32, 0.63, 1.26, 2.51, 5.02, and 10.04 mg Zn/l and 0.13, 0.26, 0.53, 1.06, 2.12, and 4.23 mg Zn/l, respectively. The ZnCl_2_ concentrations were set up based on the measured mean dissolution rate. Four replicates for each concentration and control were applied. Furthermore, a negative control (320 μl Milli-Q water) and a positive control containing NAC (160 μl Milli-Q water and 160 μl NAC suspension) were also set up.

A group of animals was randomly sampled from the stock culture into a counter filled with Milli-Q water for pure toxicity assay or filled with 10 mg/l NAC suspension for mitigation assay. From here, female specimens were selected with a pipette. Females are usually bigger and more frequent than males. The females’ vulva opening is located in the midline of the abdominal side, while the male orifice is located at the posterior end of the body, where the short, bent mating spike (spicule) can be easily recognized. Before the placement of the animals, 100 μl of Milli-Q water or NAC solution (10 mg/l concentration) was pipetted onto microplates to create a wet environment. Animals were then relocated with 2×30 μl Milli-Q water or NAC suspension into each well. Since the wells contained liquid by the time the solutions were added, the solutions with twofold concentrations were prepared before the experiments. After that, 160 μl of the test solution or Milli-Q water, in the case of the control group, was added to the test system to reach the final amount of 320 μl liquid. This way, the achieved final concentration of NAC was 5 mg/l. The microplates were incubated in a thermostat chamber under dark conditions at 20 ± 1 °C. Surviving specimens were counted after 24 h under a transmission stereomicroscope (Olympus SZH 10).

### Interaction between ZnO nanoparticles

The *P. redivivus* acute mortality test was used in the experiments investigating the interaction between nano and bulk ZnO particles. The concentration series were made from the 50–50% mixture of ZnO particles with an average size of 15 nm and 140 nm. Experiments were carried out the same way as described in “The mitigation effect of *N*-acetylcysteine and toxicity of ZnCl_2_ compared with ZnO NPs on *Panagrellus redivivus*.” In preliminary experiments, only the three highest concentrations (2.51; 5.02 and 10.04 mg/l Zn) were tested on their own and also with the addition of NAC to see if a valid result can indeed be obtained. Since there was a great difference between the toxicity of the mixture and that of the original substances, mainly with the addition of NAC, the entire experiment was repeated using the whole concentration series (0.32, 0.63; 1.26; 2.51; 5.02 and 10.04 mg/l Zn). After 24 h of incubation, the microplate was examined under a stereomicroscope. Milli-Q water was used as a negative control.

### Measuring the intracellular reactive oxygen species generation

Intracellular ROS production was measured using aminophenyl fluorescein (APF; Thermo Fisher) assay. APF is a relatively new and more specific indicator for ROS measurement than the hitherto used dyes (such as 2′,7′-dichlorodihydrofluorescein diacetate). It is more tolerant of light-induced oxidation and becomes fluorescent in the presence of hydroxyl radical (OH ·), peroxynitrite anion (ONOO–), and hypochlorite anion (OCl^−^) (Nagano 2009). APF reacts three times more strongly to hydroxyl radicals than to other ROS radicals, e.g., superoxide (·O_2_^−^), hydrogen peroxide (H_2_O_2_), or singlet oxygen (^1^O_2_) (Setsukinai et al. 2003). Several studies have suggested that the photocatalytic and antibacterial properties of nanoparticle oxides are mainly due to free and surface-bound hydroxyl radicals, although superoxide and hydrogen peroxide play a vital role in the processes (Ma et al. 2013). Thus, we can assume that OH· production is a representative of total ROS formation by ZnO NPs.

No standard guideline is available for measuring intracellular ROS with APF. Therefore, a modified method was used based on other ROS measuring methods (Wang et al. 2018, Sarasija and Norman 2018, Yoon et al. 2018). In those studies, *Caenorhabditis elegans* was the tested nematode species. The two different nematode species length is almost the same (approx. 1 mm). The diameter for *P. redivivus* on average is 50 μm (Sautter et al. 2007), while for *C. elegans*, it can vary between 45 and 80 μm (Maguire et al. 2011, Palikaras and Tavernarakis 2013, Desta et al. 2017). Therefore, *P. redivivus* was an adequate choice to replace *C. elegans* in this experiment. The measurement was set up similarly to the toxicity assay except the exposure time being only 3 h, and for all six types of compounds, three concentrations were tested: 2.51, 5.02, and 10.04 mg Zn/l (in Milli-Q water). Instead of the 96-well microplate, the test compounds were placed inside Eppendorf tubes to ensure the proper recovery of the nematode samples. After the incubation time, all replicates from one treatment were put into one tube (40 individuals/tube). Worms were suspended in 1.5 ml phosphate-buffered saline buffer (100 mM PBS, pH 7.2), the tubes were centrifuged at 600×*g* for 5 min, and then the PBS was aspirated out. Only 80 μl liquid was left inside the tubes with the animals. Subsequently, from the previously prepared APF stock (10 μM in PBS), 160 μl was immediately added to the samples. Worms were then homogenized for 2 min (Qiagen TissueLyser LT). Homogenates were centrifuged for 12 min at 17,000×*g* to separate the worm lysate from the carcass pellet (Qhaus Fe5718R). First, the protein concentration was determined from 30 μl solution (10 μl worm lysate + 20 μl APF stock) using the Bradford Assay following manufacturer’s instructions.

After that, for measuring the fluorescence, two replicates were used per treatment. Exposure was conducted in 96-well black microplate for the APF test. Seventy-five microliters of solution (25 μl worm lysate and 50 μl APF stock) was supplemented with 25 μl PBS to reach 100 μl per well. As for the blank (50 μl PBS) and the positive control (50 μl H_2_O_2_, 50 mM), 50–50 μl APF was added to each replicate. This method required an incubation of 30 min before the reading (dark condition, 37 °C). The samples were read at 490 nm (Ex)/515 nm (Em) on a microplate reader (Thermo Scientific Varioskan Lux). Measured ROS values were normalized to the mean value of the control of the given treatment. These values were also used when performing statistical analyses.

### Data analysis

Median lethal and effective concentrations (LC_50_, EC_50_) and associated confidence intervals (95% CI) were calculated by Probit analysis using the ToxRat program (Light Version 2.08) (TOXRATLIGHT2.08 n.d.). This was repeated with the Microsoft Excel Solver plug-in to verify the results (Microsoft Corporation 2010). For the statistical analysis, R Statistics 3.5.2. program was used (RCoreTeam 2013). In those experiments where assumptions were met (balanced standard deviation, normal distribution), the *p* values were calculated by one-way and two-way ANOVA (*F*) with Tukey’s honestly significant difference (*t*) post hoc test. If the conditions were not met, linear model (LM) was used when examining the relationship of concentrations to control. For comparing different curves, generalized least squares (GLS) technique and, in some cases, an interactional linear model (ILM) were used. The GLS model can also be used well for unequal variances. Normality was checked in all cases with the Shapiro-Wilk test.

## Results

### Particle characterization by SEM

**Fig. 1:**
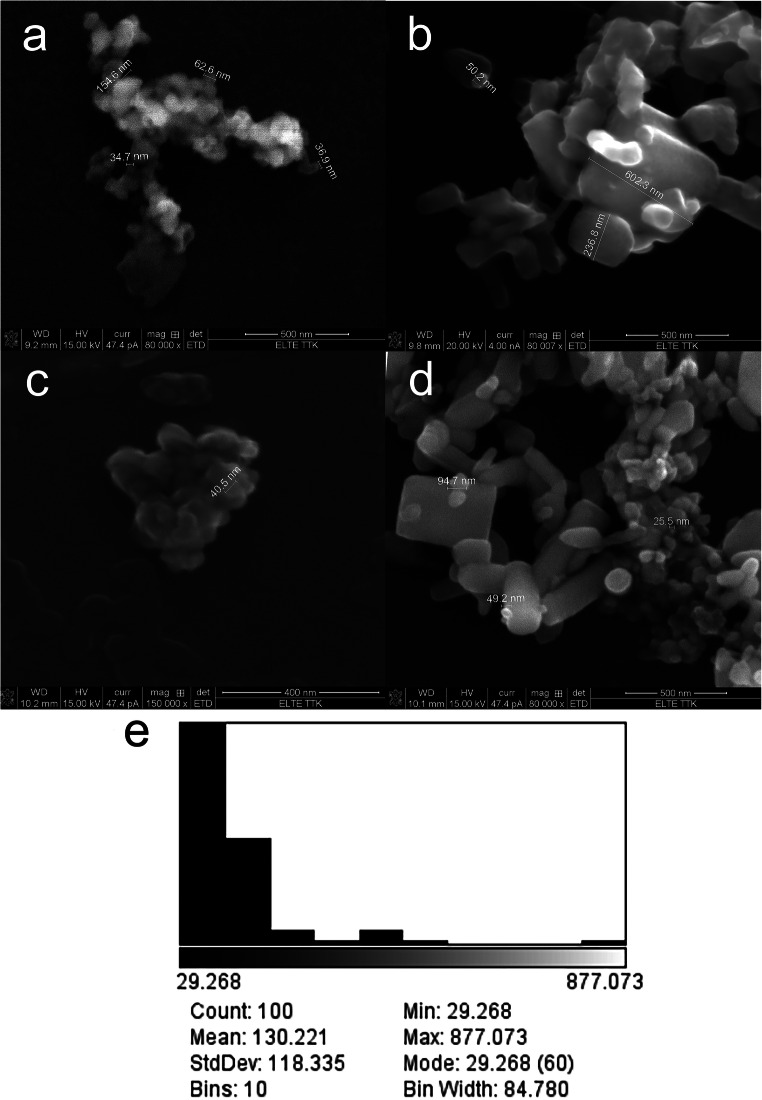
SEM images of the research compounds with NAC (15 nm ZnO (**a**), 140 nm ZnO (**b**)). SEM images of a 50–50% mixture of the two test substances individially (**c**) and with NAC (**d**). Size distribution of the mixture (**e**)

The average size for the mixture, based on measuring the diameter of 100 particles, was 130 ± 118 nm (*n* = 100). As revealed by looking at the mixture size distribution (Fig. 1e), the most common particle size by mode is 30 nm, but also larger aggregates (877 nm) may be present. In addition, as the picture clearly shows, both forms (spherical, irregular) are present in samples (Fig. 1c, d). The binary mixture of 15 nm and 140 nm ZnO individually and all three materials (15 nm, 140 nm, binary mixture) were investigated with *N*-acetylcysteine (Fig. 1). The addition of NAC did not significantly change the size or morphology of the materials (Fig. 1a, b, c, d). Coating around the materials by the NAC was not visible by SEM.

### Zinc ion dissolution

There was no significant difference in the complex Zn^2+^ dissolution associated with mitigation studies between the two pure ZnO particles of different sizes (Table 1). On the other hand, significantly more zinc ions (~30–40%) were dissolved from the binary mixture of the two substances (GLS_DF:2.6_, 15 nm, *t*=40.42, *p*<0.001; 140 nm, *t*=22.33, *p*<0.001). The measured data did not follow normal distribution (*W* = 0.80; *p* <0.05). An increase in zinc dissolution was also observed with the addition of NAC to all test materials (15 and 140 nm ZnO, 20–30%; mixture, 4%). There was a significant difference for 15 nm (GLS_DF:2.6_, *t*=−58.10, *p*<0.001) and 140 nm ZnO (GLS_DF:2.6_, *t*=−16.65, *p*<0.001) and a less prominent but also significant difference for the mixture (GLS_DF:2.6_, *t*=−4.04, *p*<0.05) compared with the dissolution values of untreated pairs of materials. Furthermore, in all cases, significant difference was found between the NAC-treated compounds (GLS_DF:2.6_, 15 nm vs. 140 nm, *t*=13.96, *p*<0.001; 15 vs. Mix, *t*=2.77, *p*<0.05; 140 nm vs. Mix, *t*=15.28, *p*<0.001). For the 15 nm and the mixture, this difference was relatively slight (~ 3%).

**Table 1:** The dissolution of 15 nm ZnO, 140 nm ZnO, and the mixture (pure and with the addition of NAC) (mg/l)

Zinc dissolution (mg/l—from 10.04 mg/l Zn–)			
	15 nm ZnO	140 nm ZnO	Mixture
Pure	4.13±0.01	4.31±0.09	5.78±0.07
Addition of NAC	5.87±0.05	5.30±0.05	6.01±0.04

### The mitigation effect of *N*-acetylcysteine and toxicity of ZnCl_2_ compared with ZnO NPs on *Panagrellus redivivus*

**Fig. 2:**
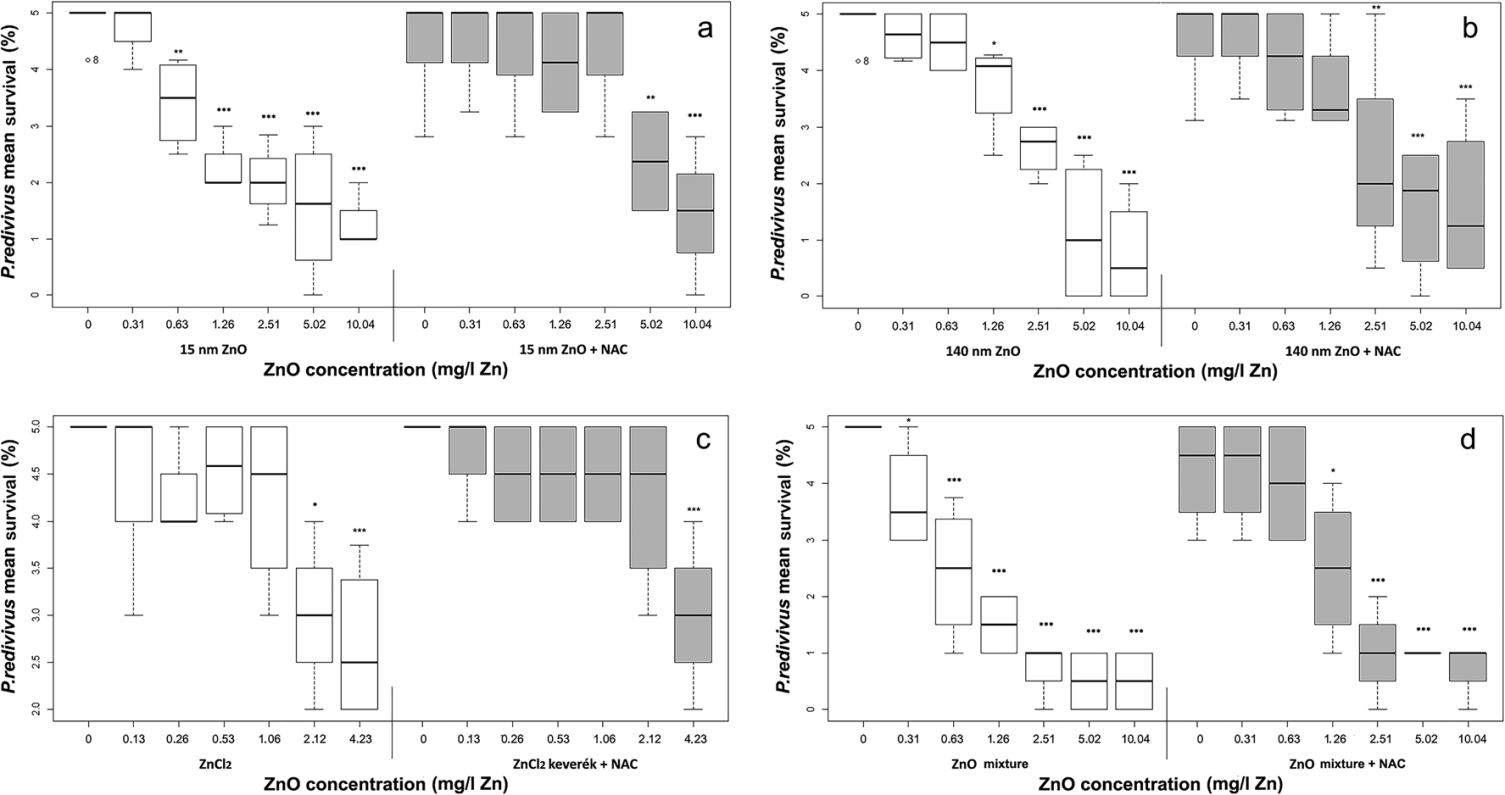
Mitigating effect of *N*-acetylcysteine on *P. redivivus* in the presence of 15 nm ZnO (**a**), 140 nm (**b**), ZnCl_2_ (**c**) and mixture of 15 nm + 140 nm ZnO (**d**) after 24 h exposure. Four replicates were used per concentration. Significance levels: *p <0.05; ***p <0.001. Linear model shows the difference between the control (0 mg/l) and the individual concentrations

The addition of NAC significantly reduced the toxic effects of 15 nm ZnO (GLS_DF:4.60_, *t*=−4.44, *p*<0.001) (Fig. 2a). In the case of 140 nm ZnO, a slight mitigating effect was observed (GLS_DF:4.56_, *t*=−3.34, *p*<0.05) (Fig. 2b). The mitigating effect showed a decreasing tendency above 2.51 mg/l and 1.26 mg/l for 15 nm and 140 nm ZnO, respectively. In contrast, when using ZnCl_2_, mitigation was only observed if the highest concentration (4.23 mg/l Zn) was excluded (LM_DF:3.44_, *t*=2.07, *p*<0.05) (Fig. 2c). The two different particle sizes were also affected differently by mitigation treatment. Notwithstanding the two highest concentrations, where none of the substances had a mitigating effect, a greater decrease in toxicity was observed in the presence of 15 nm ZnO than at 140 nm ZnO (GLS_DF:4.44_, *t*=2.09, *p*<0.05). These results are also apparent from the LC_50_ values (Table 2). Our data did not follow normal distribution (*W* = 0.85; *p* <0.05).

**Table 2:** LC_50_ values of 15 nm, 140 nm ZnO, ZnCl_2_, and the mixture on *Panagrellus redivivus*, individually and with the addition of NAC. n.d.: not determined due to the lack of data or mathematical reasons

	15 nm	140 nm	ZnCl_2_	Mixture
Pure	1.85	2.66	4.96	0.65
	(CI 95% 1.1–3.13)	(CI 95% 1.6–4.53)	(CI 95% n.d.)	(CI 95% n.d.)
Addition of NAC	14.091	10.725	5.80	1.62
	(CI 95% n.d.)	(CI 95% n.d.)	(CI 95% n.d.)	(CI 95% n.d.)

**Fig. 3:**
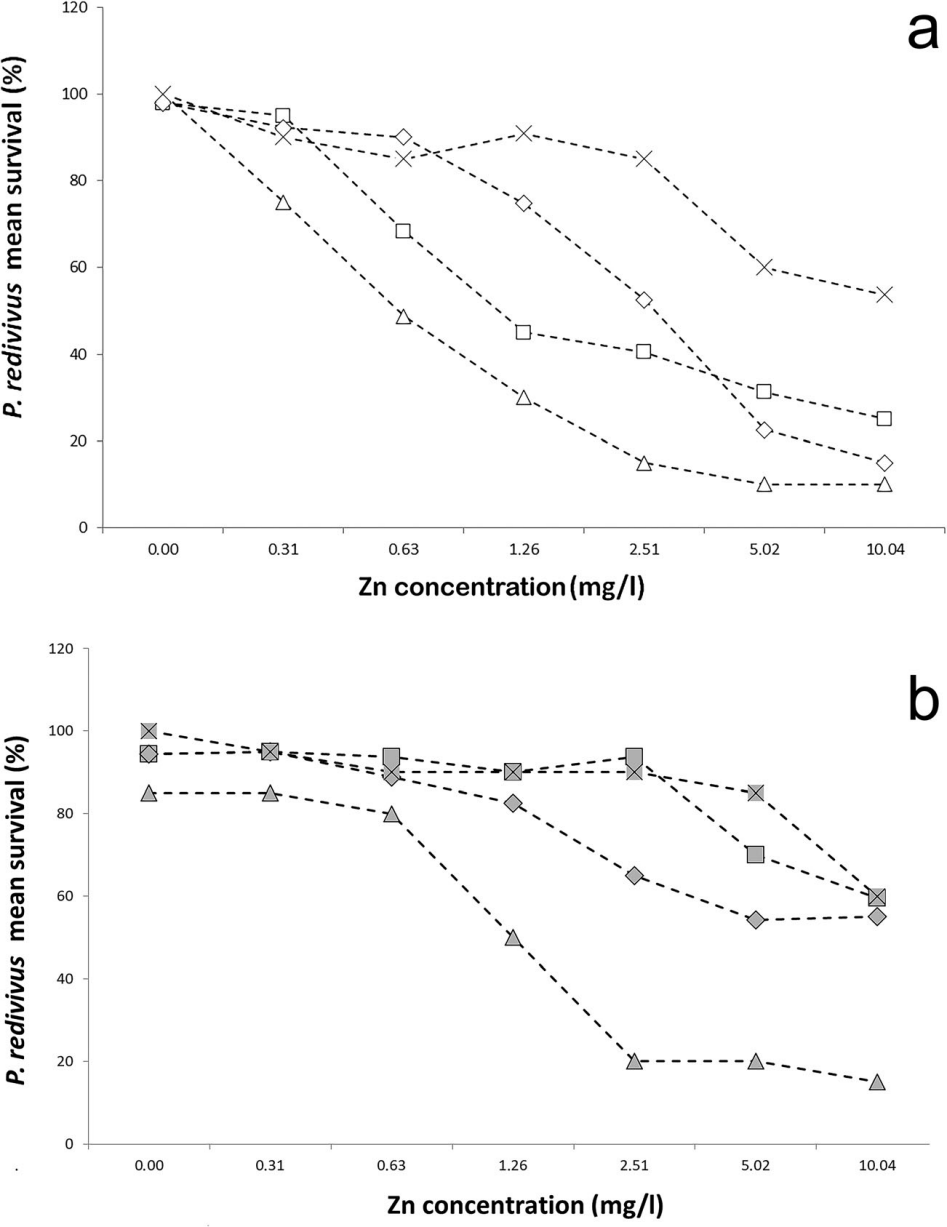
The toxic effect of 15 nm ZnO, 140 nm ZnO, ZnCl_2_, and the mixture of 15 nm + 140 nm ZnO on *P. redivivus* after 24 h exposure time without (**a** 15 nm, empty square; 140 nm, empty diamond; mixture, empty triangle; ZnCl_2_, multiplication sign) and with (**b** 15 nm, filled square; 140 nm, filled diamond; mixture, filled triangle; ZnCl_2_, squared times) the addition of NAC. In the ZnCl_2_ assay, the concentration series were set based on the dissolved ions (from 10.04 mg/l Zn; on average 4.23 mg/l Zn). The points were calculated based on the dissolution for the given concentration in the figure. Thus, for each treatment: 0.31–0.13; 0.63–0.26; 1.26–0.53; 2.51–1.06; 5.02–2.12 and 10.04–4.23 mg/l Zn. Four replicates per concentration were used

When testing the pure materials, no particle size-dependent toxicity was observed. ZnCl_2_ was significantly less toxic than the two ZnO particles in the concentration series based on the dissolved zinc content (GLS_DF:4.56_, 15 nm, *t*=−3.16, *p*<0.05; 140 nm, *t*=−3.16, *p*<0.01) (Fig. 3a).

### Interaction between ZnO nanoparticles

A synergistic increase in toxicity was observed when using the binary mixture of the two particle-sized ZnO (Fig. 3a). Compared with the 15 nm ZnO particle, the mixture proved to be significantly more toxic, even when used without the addition of NAC (GLS_DF:4.56_, 15 nm, *t*=2.05, *p*<0.05). Moreover, this difference in toxicity was further increased by the addition of NAC. Thus, in that case, the toxic effect of the mixture was stronger than both of the used ZnO size type alone (GLS_DF:4.56_, 15 nm, *t*=−3.33, *p*<0.01; 140 nm, *t*=−2.50, *p*<0.01) (Fig. 3b). There was no statistically demonstrable mitigating effect of the antioxidant on the mixture when studying all of the concentrations, although in up to 1.26 mg/l Zn, a slight decrease in toxicity was observed as compared with the mixture without NAC (GLS_DF:4.28_, *t*=−2.33, *p*<0.05) (Fig. 2d). This can also be supported by LOAEC values (pure, 0.31 mg/l; NAC, 1.26 mg/l). The mitigating effect was less evident for the mixture than for the two ZnO particles alone, as can be seen from the LC_50_ values (Table 2).

### Evaluation of reactive oxygen species generation method

The newly developed method was repeated six times to make sure it was functional. In 5 out of 6 experiments, the ROS content was between 0.374 and 0.425 μM/mg in the Milli-Q water control groups. A peak value of 0.716 μM/mg was experienced only in one case. Control values per treatment are shown in Table 3. When comparing the Milli-Q water control values, SD was 0.018 and CV 4.6%, excluding the peak value with outlier analysis by graphic representation (0.716 μM/mg) (see Appendix A for Q-Q plot).

**Table 3:** Measured ROS (μM/mg) control values

	15 nm	140 nm	Mixture
Pure	*0.698*	0.380	0.399
	*0.734*	0.374	0.420
Addition of NAC	0.399	0.425	0.425
	0.420	0.415	0.415

The peak values were indicated in italics

### Measuring the intracellular reactive oxygen species generation

**Fig. 4:**
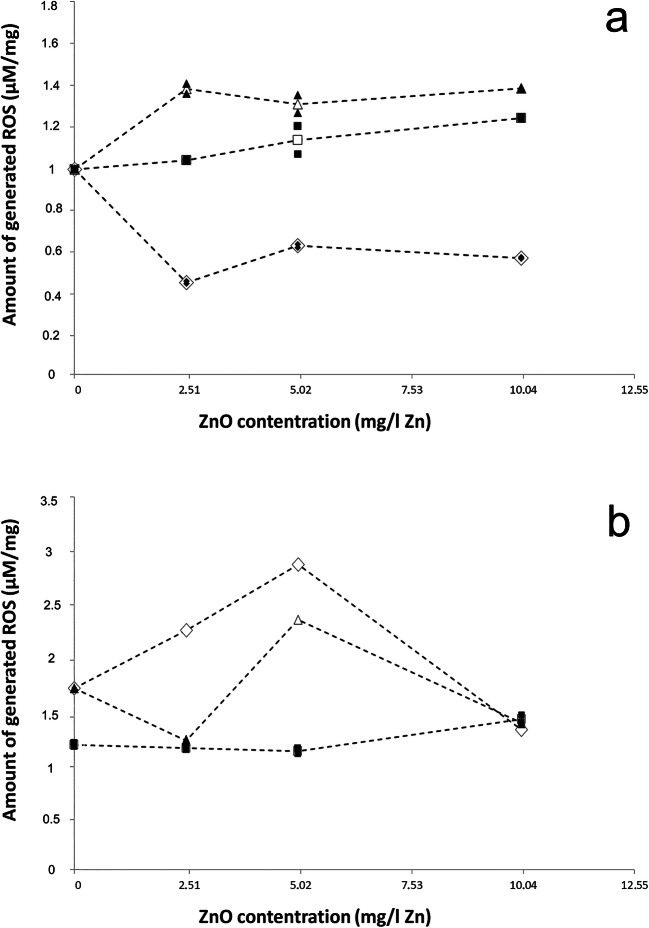
The mean amount of generated reactive oxygen radical (μM/mg) by 15 nm, 140 nm, and the mixture without (**a**) and with (**b**) the addition of NAC (15 nm, empty square; 140 nm, empty diamond; mixture, empty triangle). Two replicates per concentration were used (15 nm, filled square; 140 nm, filled diamond; mixture, filled triangle)

Among the three measured pure materials, the highest ROS production was observed for the mixture and the lowest for the 140 nm ZnO (Table 4). Therefore, 140 nm ZnO was significantly different from the mixture (LM_DF:3.8_, *t*=8.930, *p*<0.001) and 15 nm ZnO (LM_DF:3.8_, *t*=5.322, *p*<0.01) (Fig. 4a). A concentration-dependent ROS increase and a significant difference were observed in the case of 15 nm ZnO and the binary mixture (LM_DF:3.8_, *t*=4.440, *p*<0.01). In the presence of NAC, a completely different tendency was observed from when the materials were applied alone (Table 5), as there was no significant difference between the materials (Fig. 4b). Both for 140 nm ZnO and the mixture, a sharp increase in ROS production was observed at 2.51 mg/l Zn concentration, followed by a substantial decrease. For the data obtained during the reactive oxygen species measurement, a normal distribution was observed (*W* = 0.94; *p* > 0.05).

**Table 4:** Average fluorescence values of 15 nm, 140 nm ZnO, and mixture of 15 + 140 nm ZnO. Two replicates were used per measurement.. The ROS values (μM/mg) were normalized to the mean value of the control of the given treatment. Control values per treatment are shown in Table 3

	15 nm				140 nm				Mixture			
	Fluorescence (490/515 nm)	Recovery curve (50 mM H_2_O_2_)	Total protein content (μg/ml)	Normalized ROS values (μM/mg)	Fluorescence (490/515 nm)	Recovery curve (50 mM H_2_O_2_)	Total protein content (μg/ml)	Normalized ROS values (μM/mg)	Fluorescence (490/515 nm)	Recovery curve (50 mM H_2_O_2_)	Total protein content (μg/ml)	Normalized ROS values (μM/mg)
Control	2.036	41.384	25.318	1	1.549	27.996	29.300	1	1.308	25.544	25.855	1
	2.136	43.478	22.097		1.527	27.574	29.705		1.375	26.913	25.355	
10.04 mg/l Zn	1.971	40.023	25.012	1.038	1.231	22.039	65.965	0.455	1.636	32.289	24.509	1.361
	1.995	40.524	18.070	1.051	1.261	22.611	36.802	0.467	1.690	33.421	21.806	1.409
5.02 mg/l Zn	2.248	45.836	24.275	1.205	1.305	23.422	38.657	0.628	1.481	29.103	20.102	1.354
	2.008	40.792	18.222	1.073	1.336	24.004	40.608	0.643	1.392	27.278	21.878	1.269
2.51 mg/l Zn	2.058	41.845	19.880	1.248	1.264	22.658	43.338	0.582	1.626	32.082	23.541	1.383
	2.049	41.665	17.602	1.242	1.249	22.381	39.408	0.574	1.634	32.258	21.736	1.391

**Table 5:** Average fluorescence values of 15 nm, 140 nm ZnO, and mixture of 15 + 140 nm ZnO with the addition of NAC. Two replicates were used per measurement. The ROS values (μM/mg) were normalized to the mean value of the control of the given treatment. Control values per treatment are shown in Table 3

	15 nm ZnO + NAC				140 nm + NAC				Mixture + NAC			
	Fluorescence (490/515 nm)	Recovery curve (50 mM H_2_O_2_)	Total protein content (μg/ml)	Normalized ROS values (μM/mg)	Fluorescence (490/515 nm)	Recovery curve (50 mM H_2_O_2_)	Total protein content (μg/ml)	Normalized ROS values (μM/mg)	Fluorescence (490/515 nm)	Recovery curve (50 mM H_2_O_2_)	Total protein content (μg/ml)	Normalized ROS values (μM/mg)
Control	1.308	25.544	25.855	1	1.255	25.329	27.600	1	1.255	25.329	27.600	1
	1.375	26.913	25.355		1.226	24.715	20.052		1.226	24.715	20.052	
NAC control	1.604	31.633	24.849	1.224	1.763	36.108	19.853	1.730	1.763	36.108	19.853	1.730
	1.569	30.911	25.593	1.196	1.773	36.321	19.905	1.740	1.773	36.321	19.905	1.740
10.04 mg/l Zn	1.619	31.957	25.917	1.191	1.739	35.609	16.733	2.275	1.443	29.314	26.080	1.233
	1.585	31.252	26.461	1.165	0.251	4.003	13.076	0.256	1.479	30.074	19.193	1.265
5.02 mg/l Zn	1.395	27.324	24.469	1.175	2.494	51.637	18.177	2.889	1.804	36.981	14.365	2.374
	1.340	26.200	20.923	1.127	0.034	−0.596	15.859	*-*	0.039	−0.504	15.304	*-*
2.51 mg/l Zn	1.461	28.680	19.552	1.414	1.765	36.144	16.798	1.349	1.253	25.289	14.574	1.427
	1.530	30.118	20.060	1.484	0.053	−0.200	34.243	*-*	1.231	24.807	19.163	1.400

## Discussion

### The mitigation effect of *N*-acetylcysteine on Panagrellus redivivus

So far, the mitigating effect of *N*-acetylcysteine on nano-metal oxides has been demonstrated mainly in a human cell test system (Wang et al. 2014, Liu et al. 2017, El-Shorbagy et al. 2019). Wu et al. (2013) have shown the mitigation effect of NAC (5 mM) on ZnO NPs (50 μg/l) in another nematode test species. Their results were comparable with our findings. We also experienced 20% mitigation in 0.63 mg/l concentration in the case of 15 nm ZnO on the nematode *P. redivivus*, similarly as stated in Wu et al. (2013). In higher concentrations (1.26–10.04 mg/l Zn), NAC successfully reduced the toxic effects of both 15 nm and 140 nm ZnO particles on average by 50% and 30%, respectively.

Particle size-dependent mitigation is less known since bulk controls were lacking in most studies (Wang et al. 2014, Yang and Ma 2014, El-Shorbagy et al. 2019). Liu et al. (2017) compared the effects of two nano-sized ZnO particles (18.5 ± 1.2 nm and 47.1 ± 5.1 nm ZnO) on the human neuroblastoma SHSY5Y cell line. When tested alone, they found stronger toxic effects from the smaller particle size ZnO, and the addition of NAC reduced the toxicity of both particle sizes. Mitigation was the strongest at concentrations below 40 mg/l, above which a reduced effect was observed, as well as a slight difference in toxicity between the two particle sizes. In the case of the larger particle size, the mitigation was smaller, similarly to present studies, where a milder effect was observed in the case of 140 nm ZnO. These results are supported by our earlier findings where we found 140 nm ZnO to be more toxic in the presence of soil solution (to the nematode *P. redivivus*) and artificial soil (to the springtail *Folsomia candida*) (Kiss et al. 2018). From the two substances applied in our study, the larger particle size toxicity was more difficult to mitigate by the NAC. This is presumably because the two materials had different surface charge densities, distributions, and electrical potentials due to their different size (Abbas et al. 2008, Holmberg et al. 2013) as well as different morphology (Andelman 1995).

Liu et al. (2017) also compared the effects of nano and bulk ZnO with ZnCl_2_ similarly to our experiments. Although mitigation was observed in this case at two lower concentrations (122.9 μM and 245.7 μM ZnCl_2_), above this, cell survival rate decreased below 10% even with the addition of NAC. Therefore, NAC had a much weaker effect on ZnCl_2_ than on the nanoforms, similarly to our results. According to the present experiments, NAC has a lower effect on ionic toxicity.

No specific reference has been found regarding the effect of *N*-acetylcysteine on the dissolution of Zn, but the chelating properties of the material have been described in several studies (Rossignol 2005, Flora and Pachauri 2010, Giampreti et al. 2016). Solubility values in the present study were significantly higher when NAC was added, as here the total Zn content was measured. While from the two tested ZnO particles alone, approximately the same amount of Zn dissolved, after the addition of NAC, significantly lower values were obtained from ZnO with the larger particle size. Our experiments have shown that *N*-acetylcysteine addition reduces the toxicity of 15 and 140 nm ZnO particles on *P. redivivus*.

Based on our test results, NAC can be favorable as mitigation agent; however, before the environmental applications, the effects of NAC require more research with relevant test systems.

### Elucidate the role of ionic toxicity in the toxicity mechanism of ZnO NPs

The concentrations of ZnCl_2_ used in these experiments represented the amount of dissolved Zn ions present in the test system. It can be seen that behind the toxic effects of ZnO particles, there could be additional properties apart from the toxicity of dissolved ions, like spontaneous and size relevant ROS generation and interaction between the cell and the particle. These results are in agreement with the findings of Song et al. (2010) who showed that the amount of Zn^2+^ dissolved and the amount of ROS generated could not induce the degree of cytotoxicity that was observed. Thus, it is assumed that additional toxicity factors must be present to produce the observed effects.

### Synergistic toxicity increases due to mixing the ZnO particles

Studies have also shown an increase in synergistic toxicity when mixing two different nanomaterials, e.g., ZnO NP + AG NP (Jafari et al. 2011), Au NP+ Pt NP (Mott et al. 2007), and Ag NP + TiO_2_ NP (Li et al. 2011). However, a reduction in toxicity was detected when nanoparticulate ZnO and TiO_2_ were mixed, and this effect was explained by the Zn^2+^ adsorption on the TiO_2_ surface and thus became less available for test animals (Tong et al. 2014). In the present study, the binary mixture of two different particle sizes ZnO showed a substantial increase in toxicity, in the Zn dissolution rate and the ROS production. The increase in toxicity was also observed with the addition of *N*-acetylcysteine, as the lowest mitigating effect was detected in the case of the mixture. There is no literature available on testing mixture toxicity with the addition of NAC. According to Liu et al. (2017) and the present experiments with NAC, the antioxidant has less influence on the toxic effects caused by dissolved ions. The measured amount of dissolved ions was 1.5 times higher in the mixture than in the two substances individually. This could be one reason behind the milder mitigation effect on the mixture.

SEM images of the mixture compared with the pure compounds images (Kiss et al. 2018) confirm that both materials are present in the new mixture and a new ZnO with an average particle size between the two other materials (130 ± 118 nm), with both spherical and irregular particles, has been generated. Particle distributions also show that approximately 30% of the particles found in the 140 nm ZnO are in the nano-size range (37–97 nm, most often 37 nm) (Kiss et al. 2018), so the ratio of nanoparticles has increased above 50% due to the mixing of the two materials. Moreover, larger particles are believed to have a dispersing effect on the test system. In the mixture, particles aggregated more with each other—due to the different charge distribution—than with particles of their size group. As a result, small particles aggregated on the surface of 140 nm ZnO particles fixed the large surface, increasing dissolution, reactivity, and thus toxicity. This is also probably because of the different surface properties (Abbas et al. 2008, Holmberg et al. 2013) and morphology (Andelman 1995). Therefore, by mixing the particles, the different adverse effects of the two materials (15 nm—particle size, 140 nm—irregular morphology) reinforce each other, which may be the reason for the stronger toxic effects.

### Evaluation of reactive oxygen species generation method

The method suggested by literature proved to be unsuitable for measuring the produced ROS in our test system. Sarasija and Norman (2018) recommend removing supernatant from above the animal with 100 μl of liquid remaining in the tubes. This is not feasible since the amount of liquid remaining in the Eppendorf tube cannot be accurately determined. Even though animals settle to the bottom of the tube, for such a small amount, it is inevitable to enter the pipette. Yoon et al. (2018), on the other hand, suggests a more feasible method by pipetting the animals onto a glass slide. However, the recommended volume of 10 μl was very low for the 40 animals used in our experiments. As based on our own experience, the animals can be moved with 80 μl of liquid. In both protocols and Wang et al. (2018) suggest adding an indicator to the sample after lysis, but in our tests, it was found that too much time elapses between incubation and measurement. Consequently, animals still alive can degrade the produced reactive oxygen species. The APF was added immediately after the test end and lysed with, thus preventing the breakdown of ROS. After these changes, the method became usable, and protein and fluorescence measurements of the samples were successful.

During the method development, we managed to modify the ROS measurements described by Sarasija and Norman (2018), Wang et al. (2018), and Yoon et al. (2018), so they would be usable and reproducible with the test species and indicator material used in the present study. Without improvements, we failed to use the method. Subsequently, we applied the refined method successfully several times. In the future, it can be used to measure the induced intracellular reactive oxygen species with APF indicator in the *P. redivivus* and probably in comparable test species.

### Testing reactive oxygen species production

Concentration-dependent production of reactive oxygen species is generally detected in ZnO NPs assays (Xia et al. 2008, Song et al. 2010, Liu et al. 2017, Huang et al. 2019). Compared with other metal oxides, H_2_O_2_ production is medium dependent, but superoxide production was always the highest in the case of ZnO NPs (Xia et al. 2008). In most instances, comparisons of nano and bulk ZnO were found to have a visible particle size-dependent effect; ZnO NPs induced higher ROS generation (Song et al. 2010, Liu et al. 2017) than their bulk counterparts. In the present experiments, the highest amount produced was found after 3 h of exposure time in the mixture, and the lowest appeared in the 140 nm ZnO. When NAC was added, ROS were produced to a much greater extent in the case of 140 nm and the mixture than independently. In literature, ROS production is generally reduced by NAC (Wang et al. 2014). It is hypothesized that NAC-induced GSH production has not yet begun during the 3-h ROS exposure (Farbiszewski et al. 2000), so the decrease was not studied. Further experiments should be performed with longer exposure times (6 and 12 h).

### Summary of discussion

*P. redivivus* was sensitive to both 15 and 140 nm ZnO treatment. Our studies have shown that *N*-acetylcysteine can mitigate the toxic effects of both studied particle sizes. From the applied two particle sizes, 140 nm ZnO toxicity was found to be harder to mitigate by NAC. This can be explained by morphological differences (Iswarya et al. 2015, Tong et al. 2015), the difference in charge distribution (Andelman 1995), and the fact that *N*-acetylcysteine was less able to make complexes with this material than with smaller particle-sized one, likely also due to morphological differences. The NAC had less mitigation on the toxic effect of zinc ions (Liu et al. 2017). This was possibly one of the reasons why lower mitigating effects were found in the case of the mixture of the two substances, where the solubility was significantly higher. The ZnCl_2_ concentrations were used to represent the amount of dissolved Zn ions present in the test system, which proved that other particle size-dependent toxic effects are also important in ZnO NP toxicity besides dissolution. Previous findings (Song et al. 2010) are supported by our results. When the two materials were applied in binary mixtures, the toxic effects increased significantly. Besides, the dissolved zinc content and the ROS generation also increased. It is assumed that the chemical and physical properties of the materials (several smaller particles—higher bioavailability, increased toxicity from a fixed, large surface area, morphological aspects) have been mutually reinforcing each other to form a much more reactive mixture that is more toxic to *P. redivivus* test organism. Studies have shown that 15 nm ZnO alone can generate higher amounts of ROS and dissolved ions than 140 nm ZnO.

On the other hand, such different trends in toxicity can be changed by influencing some parameters of the test system in order to neutralize the toxic effects of 15 nm ZnO. This happened in present study when adding NAC as a mitigation agent or in Kiss et al. (2018) when soil solution as an alternative test media was applied. In both cases, 140 nm ZnO will immediately become more toxic than its counterpart with a smaller size. This is presumably due to the irregular particle morphology. ROS production was induced by all used materials measured by the modified method. When testing the substances by themselves for ROS production, it was the highest in the mixture and the lowest in the 140 nm ZnO. With the addition of NAC, due to the low toxicity of the substance itself, the control ROS values were somewhat higher than with the individually tested compounds. Higher exposure times are required for the assay for the substance to exert its effect (Farbiszewski et al. 2000).

## Conclusion

Our findings testify the need to investigate the mechanism behind ZnO nanoparticles toxicity. Toxicity mitigation by special antioxidants is a new way to decrease the environmental risk of nanoparticles. As proved by our study, *N*-acetylcysteine can mitigate the effect of ZnO NPs on nematodes. It is also important to investigate the same compound in different test systems, as only one way of testing can lead to false assumptions. In the present study, we only experienced size related toxicity difference with the addition of NAC. Moreover, our findings highlight the role of dissolution unrelated ROS production in toxicity. Our study suggests taking into consideration the interaction between compounds as a hazard and risk assessment for nanomaterials. Future studies need to focus more on morphology and charge density distribution of the researched nanomaterials.

## Supplementary information

ESM 1 (JPG 480 kb)

## Data Availability

The data that support the findings of this study are available online (10.6084/m9.figshare.12933284.v1) and from the corresponding author (lolavirag.kiss@gmail.com).
