# Supplementary figures and images for: Toxicity mitigation by N-acetylcysteine and synergistic toxic effect of nano and bulk ZnO to Panagrellus redivivus
Source: Environ Sci Pollut Res Int. 2021 Mar 2;28(26):34436–49. doi: 10.1007/s11356-021-12674-7 (PMC8275494; doi:10.1007/s11356-021-12674-7)

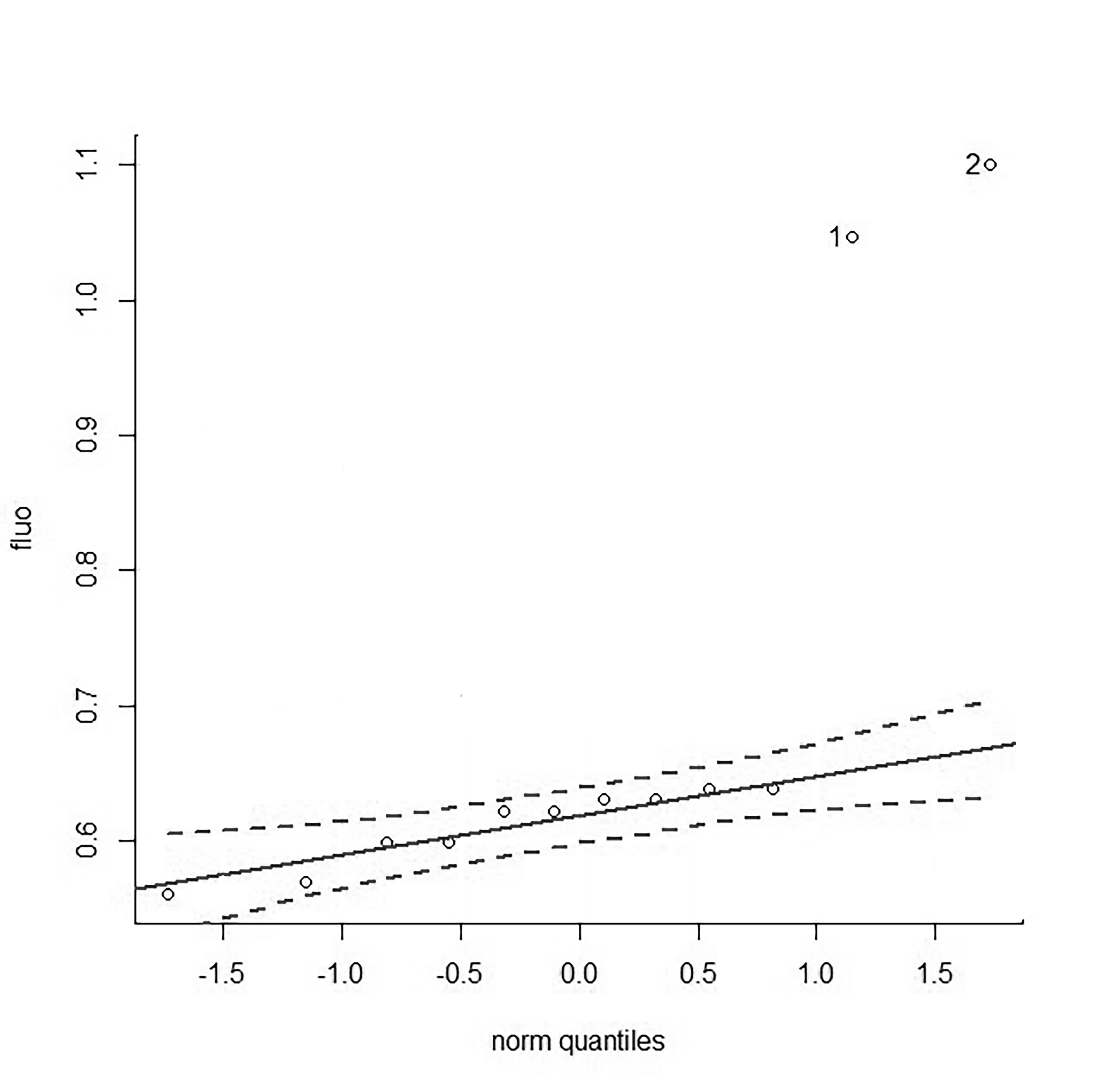

Supplement: Supplementary file 1 — (JPG 480 kb) [file 11356_2021_12674_MOESM1_ESM.jpg]
